# Seasonality modulates the direct and indirect influences of forest cover on larval anopheline assemblages in western Amazônia

**DOI:** 10.1038/s41598-021-92217-9

**Published:** 2021-06-16

**Authors:** Adriano Nobre Arcos, Francisco Valente-Neto, Francisco Augusto da Silva Ferreira, Fábio Padilha Bolzan, Hillândia Brandão da Cunha, Wanderli Pedro Tadei, Robert M. Hughes, Fabio de Oliveira Roque

**Affiliations:** 1 Programa de Pós-Graduação em Ecologia e Conservação, Instituto de Biociências, Universidade Federal de Mato Grosso do Sul (UFMS), Campo Grande, MS Brazil; 2 Programa de Pós-Graduação em Ciências Biológicas - Entomologia, Instituto Nacional de Pesquisas da Amazônia (INPA), Manaus, AM Brazil; 3 Programa de Grande Escala da Biosfera-Atmosfera na Amazônia (LBA), Laboratório de Química Ambiental, Coordenação de Dinâmica Ambiental, Instituto Nacional de Pesquisas da Amazônia (INPA), Manaus, AM Brazil; 4 Laboratório de Malária e Dengue, Coordenação de Sociedade, Ambiente E Saúde, Instituto Nacional de Pesquisas da Amazônia (INPA), Manaus, AM Brazil; 5 Amnis Opes Institute, Corvallis, OR USA; 6 Department of Fisheries & Wildlife, Oregon State University, Corvallis, OR USA; 7 Centre for Tropical Environmental and Sustainability Science (TESS), James Cook University, Cairns, Australia

**Keywords:** Entomology, Ecology, Ecological modelling

## Abstract

Serious concerns have arisen regarding urbanization processes in western Amazônia, which result in the creation of artificial habitats, promoting the colonization of malaria vectors. We used structural equation modelling to investigate direct and indirect effects of forest cover on larval habitats and anopheline assemblages in different seasons. We found 3474 larvae in the dry season and 6603 in the rainy season, totalling ten species and confirming the presence of malaria vectors across all sites. Forest cover had direct and indirect (through limnological variables) effects on the composition of larval anopheline assemblages in the rainy season. However, during the dry season, forest cover directly affected larval distribution and habitat variables (with no indirect affects). Additionally, artificial larval habitats promote ideal conditions for malaria vectors in Amazonia, mainly during the rainy season, with positive consequences for anopheline assemblages. Therefore, the application of integrated management can be carried out during both seasons. However, we suggest that the dry season is the optimal time because larval habitats are more limited, smaller in volume and more accessible for applying vector control techniques.

## Introduction

Urbanization, loss of native vegetation and habitat modification have dramatically altered tropical forests globally^1,2^. These land use changes have led to losses of biodiversity and ecosystem services^3,4^ and affected the population dynamics of vector mosquitoes with important consequences for public health^5,6^. Mosquitoes are key vectors of human diseases, globally transmitting more than 17% of all infectious diseases. Dengue and malaria cause 440,000 deaths annually, and the large numbers of people infected often overloads healthcare systems^7^. In Brazil, >99% of malaria cases occur in Amazônia, including 63,361 cases of malaria in the state of Amazonas in 2019^8,9^.

Serious concerns have been raised about urbanization in western Amazônia^10^, one of the world’s richest biodiversity regions, which houses more than 7,400,000 humans^11^. Urbanization and human expansion in the region have been increasing both in urban centers and in peri-urban areas^12,13^. The relationship between deforestation and malaria dynamics is complex and interconnected. For example, deforestation and urbanization increase the number and distribution of habitats available for the malaria vector *Anopheles darlingi* Root, 1926, thereby expanding malaria transmission^6^. These anthropogenic impacts also increase malaria cases resulting from increased contacts between humans and vector species^14^. In contrast, high numbers of malaria cases reduce deforestation through socio-economic mechanisms^15^.

Disentangling the role of several mechanisms by which deforestation affects mosquito diversity, distribution and abundance in tropical forests is challenging, because forest losses likely have direct and indirect effects on larvae and adults. For example, forest loss can reduce anophelines in the subgenus *Kerteszia*, which depend on tree holes and bromeliads for larval habitats^16^. The loss of native vegetation can also change microclimatic conditions, such as temperature and humidity, which in turn may affect mosquito population dynamics^17^. Land use changes may also influence mosquito species by reducing their host taxa, such as non-human primates, thereby reducing pathogen transmission^18^.

Deforestation and urbanization processes often create new artificial larval habitats, such as trash, dams, ponds, and clay pits, promoting colonization by mosquitoes, including malaria vectors in Amazônia^19,20^. The amount of forest around artificial habitats also influences water quality variables, such as temperature, dissolved oxygen, sediments, and dissolved and suspended organic matter. Seasonality can also modulate water quality^21^. For example, in the dry season, lentic habitats are reduced, leading to increased primary production and dissolved solids and decreased pH, which are positively correlated with the presence of *Anopheles* species in aquatic systems^22,23^. Therefore, distinguishing the direct (mediated only by forest loss) and indirect (forest loss effects on larval habitats) effects of forest loss on mosquito diversity and abundance is fundamental for understanding and predicting mosquito assemblages.

**Figure 1:**
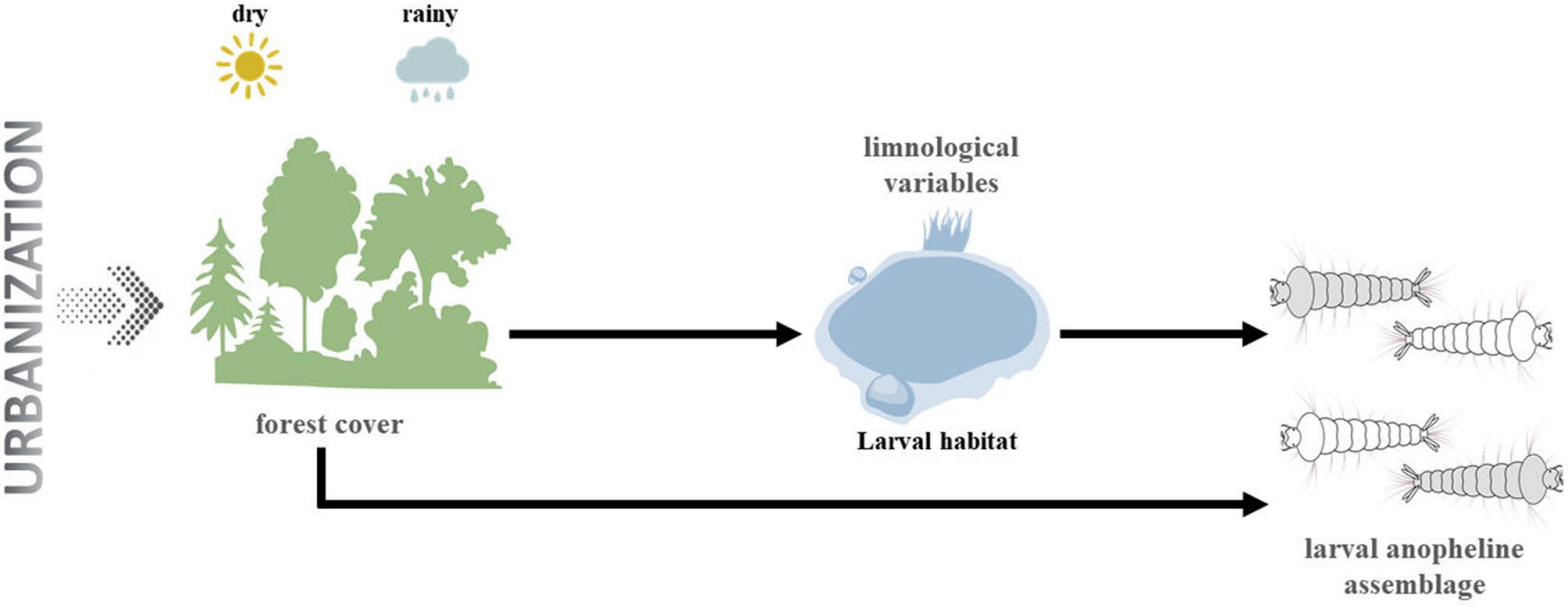
Conceptual structural equation model. The image was created using Abode Illustrator 2020 (https://www.adobe.com/br/products/illustrator/).

In this study we assessed how forest loss might directly and indirectly (through limnological variables) affect *Anopheles* assemblages in Manaus, Amazonas. We hypothesized that forest loss and limnological variables would affect mosquito assemblages in the rainy and dry seasons differently because of their differing effects on mosquito habitat conditions (Fig. 1). We expected that the forest cover gradient and limnological variables would have stronger effects on larval assemblages during the dry season. Forests play a critical role in retaining moisture (including larval habitats) and filter some forest dependent species. Also, during the dry season, water levels are decreased and organic matter is concentrated, strengthening effects of limnological variables on mosquito assemblages.

## Materials and methods

### Study area

**Figure 2:**
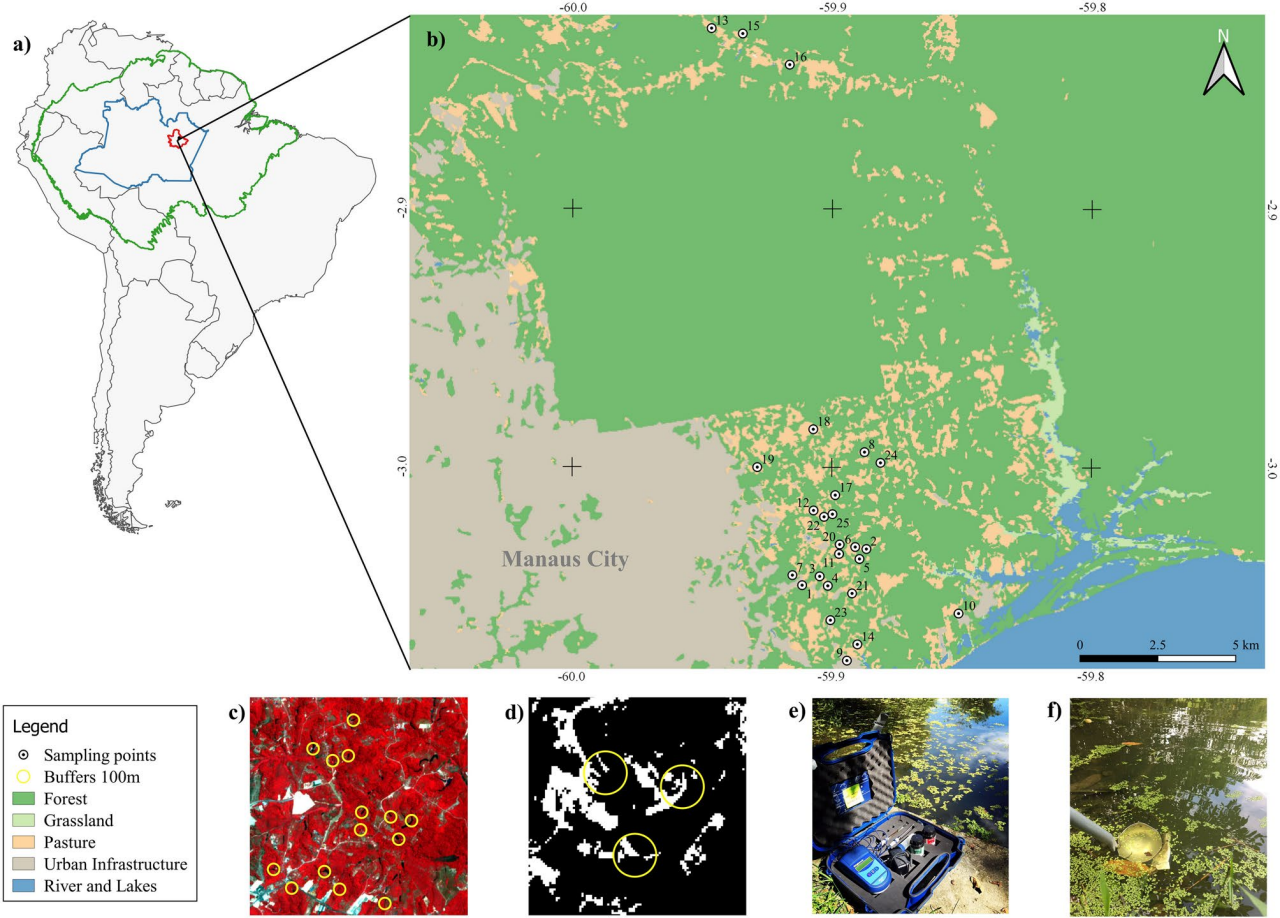
Locations of artificial larval *Anopheles* habitats in the Manaus periurban area. (**a**) Amazon River basin with the delineation of Amazonas state and Manaus; (**b**) sites, cover and land use; (**c**) composition of bands 8-4-3 for vegetation classification; (**d**) forest cover in 100 m buffers; (**e**) collection of limnological variables; (**f**) standard ladle for collecting larvae. The map was created using QGIS 3.16.5 (https://download.qgis.org).

Manaus, the capital of Amazonas, has an area of 11,401,092 km^2^ and an estimated population of 2,182,763 inhabitants^11^. It is located in central Amazônia, the world’s largest tropical forest. The region has two seasons, a December-May rainy season with high volumes of rain (~30 cm per month) and a June-November dry season with little rain (~6 cm per month)^24^ (Fig. 2).

### Landscape analysis

We built non-overlapping buffers of 100 m in radius around 25 larval habitats that were created by human modifications (e.g., clay pits and ponds)^20^ and estimated the proportion of forest within each buffer (Fig. 2; Supplementary Table S1). Biological knowledge of species dispersal are used to support the spatial extent (buffer size) used in ecological and entomological studies^25,26^. Nonetheless, knowledge of dispersal movements of anopheline species is limited, so we used 100 m radii to avoid overlapping between neighbouring buffers. Moreover, 100 m represents an approximate mean of dispersion movement for some species^27^, which is important for relating dispersion of adult *Anopheles* from their larval habitats^28^. To classify land use, we used October 2016 images from the Sentinel-2 Level-1C sensor with a 10-m spatial resolution^29^. After processing the images, bands 3, 4 and 8 (green, red and near infrared respectively) were merged to perform a semi-automatic classification in Quantun GIS version 3.4.13 - Madeira, using the Plugin SCP (Semi automatic classification plugin) version 6.4.0 of Greenbelt. The classification resulted in binary data (e.g., forest or non-forest) and classification accuracy was tested via the SCP Plugin in Quantum GIS using Google Earth images as references.

### Anopheles larval sampling

Larvae were collected in 2015 and 2016 one time each season for 30 min at each site by using a standard ladle with 350 mL volumetric capacity and a 1-m cable (Fig. 2 f). The larvae were fixed in McGregor solution and sent to the Laboratório de Malária e Dengue of the Instituto Nacional de Pesquisas da Amazônia (INPA) for identification. Collections were authorized under SISBIO permit 21264/5 and larvae were identified by using taxonomic keys^30–33^.

### Abiotic variables

Water samples were collected in sterile flasks and sent to the Laboratório de Química Ambiental (INPA), for filtering, drying and weighing total suspended solids^34^. At each site, we used portable Orion pH 290A and YSI dissolved oxygen meters to measure pH, dissolved oxygen and water temperature in situ (Supplementary Fig. S1) (Fig. 2e). Daily precipitation amounts (mm) were obtained from the Manaus automatic meteorological station (BDMEP A101) of the National Institute of Meteorology^35^.

### Data analyses

To compare rainfall (mm) between seasons, we first run a Shapiro-Wilk normality to select an appropriate analysis. Based on the non-normality of the data, we used the Kruskal-Wallis test. We also used two ordination techniques to summarize both environmental variables and anopheline larval assemblage composition. To summarize, the variance of environmental variables in a reduced space, we used Principal component analysis (PCA). The scores of the first two PCA axes that captured most of variation were used to depict gradients in environmental variables (predictors). Principal coordinate analysis (PCoA) was used to summarize anopheline larval composition into a low dimensional space. Unlike PCA, which preserves Euclidean distance between objects, PCoA ordinates objects on the basis of any resemblance index, which is more aproproate to count data. We used Hellinger distance as a dissimilarity measure of assemblage composition, which produces good representation of objects in ordination techiniques^36^. The scores of the two PCoA axes were used to represent variation in anopheline larval composition (response variables).

To compare rainfall (mm) between seasons, we used the Kruskal-Wallis test. The choice of this non-parametric analysis was based on the Shapiro-Wilk normality test. We also used two ordination techniques to summarize both environmental variables and anopheline larval assemblage composition. Principal component analysis (PCA) was used to summarize, in a low-dimension space, the variance of environmental variables into orthogonal axes; the scores of the first two PCA axes were used to depict gradients in environmental variables (predictors). We used principal coordinate analysis (PCoA) to summarize anopheline larval assemblage composition into orthogonal axes. Unlike PCA, which preserves Euclidean distance between objects, PCoA ordinates objects on the basis of any dissimilarity, allowing more flexible handling of ecological data, such as counts. We used Hellinger distance as a dissimilarity measure of assemblage composition, which is ideal for linear models. The scores of two PCoA axes were used as response variables.

Percent forest cover, PCA and PCoA axes were used to create a causal model (Fig. 1) that was tested using structural equation modeling (SEM)^37^. SEM is a useful framework for revealing causal relationships between predictor and response variables, explicitly including theory *a priori* (Fig. 1)^38^. In this framework, we compared patterns in the data to those implied by the *a priori* model, seeking to minimize difference between the model predictions and observed data. All relationships were modelled using Gaussian linear relationships. We used the maximum likelihood chi-square formula and the associated p-value to test model adequacy. The model would be considered suitable for our data when p > 0.05. Individual path coefficients were assessed using z-scores and p-values. The analyzes were performed in R, using the Lavaan package^39,40^.

## Results

In the dry season, we collected 3474 individuals and 6603 individuals were collected in the rainy season. The most abundant species in both seasons were *A. triannualtus* (45.9 and 40.6%), *A. darlingi* (27.8 and 28.7%) and *A. nuneztovari* (9.4 and 13.5%) respectively. The malária vector, *A. darlingi,* was present in all larval habitats (Table 1).

**Table 1:** Seasonal abundance distribution of *Anopheles* larvae in 25 larval habitats in the Manaus periurban area.

Species	Abundance (%)	
	Dry	Rainy
*A. (Nyssorhynchus) triannulatus* (Neiva & Pinto, 1922)	1595 (46%)	2686 (41%)
*A. (Nyssorhynchus) darlingi* Root, 1926	971 (28%)	1899 (29%)
*A. (Nyssorhynchus) nuneztovari* Gabaldon, 1940	330 (9%)	894 (14%)
*A. (Nyssorhynchus) albitarsis* s.l. Lynch-Arribálzaga, 1878	151 (4%)	350 (5%)
*A. (Stethomyia) nimbus* (Theobald, 1902)	209 (6%)	315 (5%)
*A. (Anopheles) peryassui* Dyar & Knab, 1908	121 (3%)	212 (3%)
*A. (Nyssorhynchus) braziliensis* (Chagas, 1907)	45 (1%)	119 (2%)
*A. (Nyssorhynchus) oswaldoi* (Peryassú, 1922)	38 (1%)	75 (1%)
*A. (Nyssorhynchus) evansae* (Brèthes, 1926)	14 (0.4%)	46 (0.6%)
*A. (Nyssorhynchus) deaneorum* Rosa-Freitas, 1989	0 (0%)	7 (0.1%)
Total	3474	6603

Daily rainfall in the dry season ranged from 10.7 to 31.3 mm (mean = 26.6 ± 8.5 mm standard deviation) and in the rainy season, rainfall ranged from 235.3 to 303.9 mm (mean = 275.7 ± 29.7 standard deviation), indicating a significant seasonal difference in rainfall (Kruskal-Wallis, x^2^ = 37.857, df = 1, p<0.0001) (Supplementary Fig. S2).

**Figure 3:**
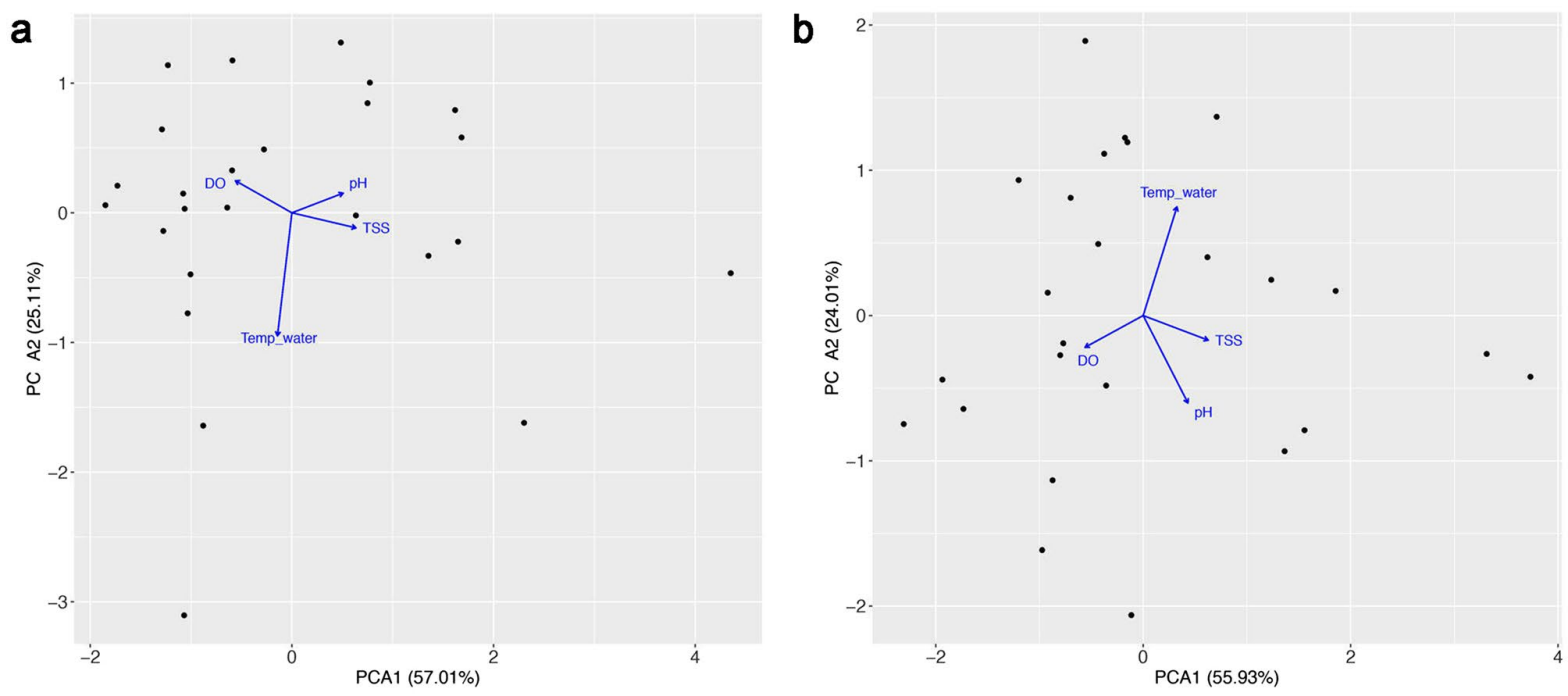
PCA of the limnological variables sampled in 25 sites in Manaus during the dry (a) and rainy (b) seasons. DO: dissolved oxygen; TEMP_WATER: water temperature; TSS: total suspended solids; Black points are sites.

The first two PCA axes explained 82% of the variation in water quality variables in the dry season and 80% in the rainy season. In both seasons, PCA1 captured a gradient of water quality, from sites with high dissolved oxygen concentration (negatively associated with PCA1) to those with high total suspended solids and pH (positively associated with PCA1). The most important PCA2 variable was water temperature (negatively associated in the dry season, positively associated in the rainy season) (Fig. 3).

**Figure 4:**
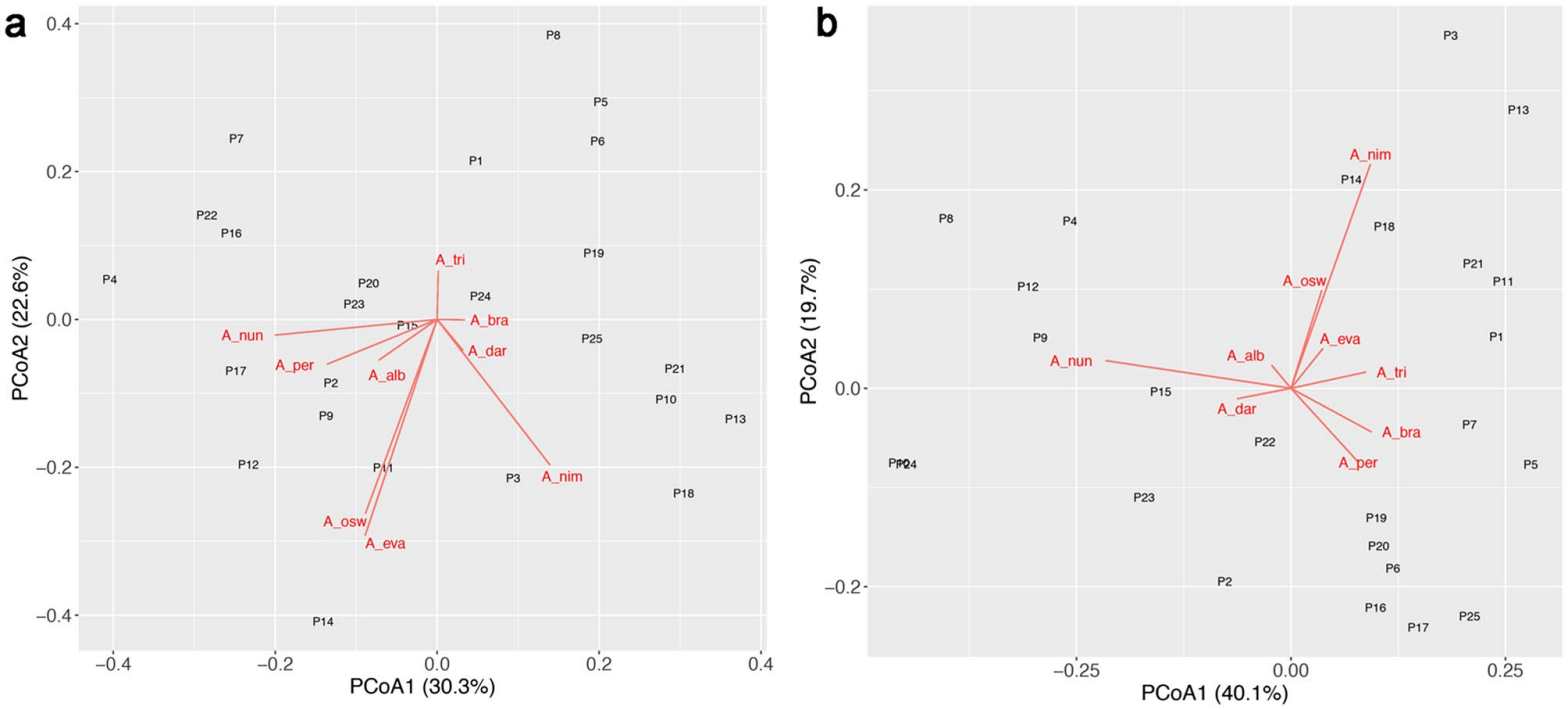
PCoA of anopheline larval assemblage composition in 25 sites in Manaus during the dry (**a**) and rainy (**b**) season. A_nun: *Anopheles nuneztovari*; A_per: *Anopheles peryassui*; A_alb: *Anopheles albitarsis*; A_osw: *Anopheles oswaldoi*; A_eva: *Anopheles evansae*; A_tri: *Anopheles triannulatus*; A_bra: *Anopheles braziliensis*; A_dar: *Anopheles darlingi*; A_nim: *Anopheles nimbus*; Numbers are sites.

The first two PCoA axes explained 52% of the variation in anopheline assemblages in the dry season and 60% in the rainy season. The most important species on the first axis in the dry season were *A. nuneztovari* (negatively associated) and *A. nimbus* (positively associated). In the rainy season, *A. nuneztovari* was also negatively associated with PCoA1, but *A. peryassui*, *A. nimbus*, *A. braziliensis* and *A. triannulatus* were positively associated with this axis. The most important species associated with PCoA2 in the dry season were *A. oswaldoi*, *A. evansae*, and *A. nimbus* (all negatively associated), but in the rainy season the most important species was *A. nimbus* (positively associated) (Fig. 4). Thus, there was a clear seasonal difference in anopheline larval assemblages.

**Figure 5:**
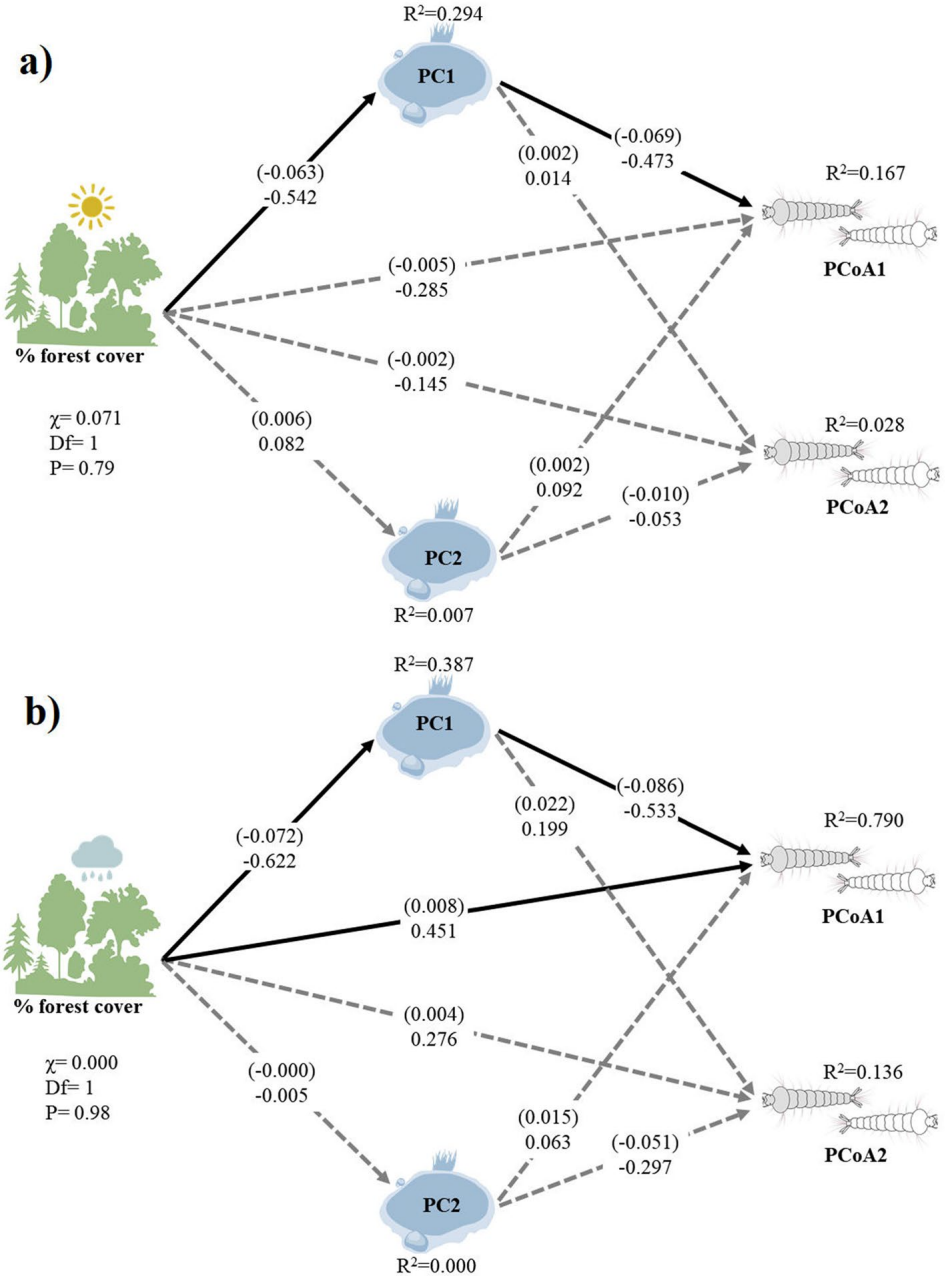
Schematic representation of dry **(a)** and rainy **(b)** season SEM results. The continuous arrows indicate significant paths and the dashed arrows indicate insignificant paths; PC1 and PC2 are the scores of the ordination of site water quality variables and PCoA1 and PCoA2 represent larval anopheline assemblage composition captured by PCoA. R2 values are reported for each endogenous variable and non-standardized and standardized (in parentheses) coefficients are indicated on each path. The image was created using Abode Illustrator 2020 (https://www.adobe.com/br/products/illustrator/).

In both seasons, the SEMs supported our hypotheses (dry season: χ = 0.071; df = 1; p = 0.79; rainy season: χ = 0.000; df = 1; p = 0.98) (Fig. 5). Forest cover negatively affected PC1 in the dry season (coefficient= − 0.542, z= − 3.227, p= 0.001, R2 = 0.294), meaning that increased forest cover was associated with decreased values along PC1 (sites with more dissolved oxygen). PC1 was negatively associated with PCoA1 (coefficient= − 0.473, z= − 2.179, p= 0.029), but did not affect PCoA2 (coefficient= 0.014, z= 0.062, p= 0.951). The PCoA1-PCA1 relationship means that greater levels of total suspended solids and pH were associated with increased numbers of *A. nuneztovati*, *A. peryassui*, *A. albitarsis*, whereas higher dissolved oxygen levels were associated with increased abundance of *A. nimbus*. The direct effect of forest cover on PCoA1 was insignificant (coefficient= − 0.285, z= − 1.307, p= 0.191), and the indirect effect of forest cover via PC1 was marginally significant (coefficient= 0.264, z= 1.834, p= 0.067). Forest cover did not affect PC2 (coefficient= 0.082, z= 0.414, p= 0.679), and PC2 did not affected PCoA1 (coefficient= 0.092, z= 0.500, p= 0.617) or PCoA2 (coefficient= 0.053, z= − 0.269, p= 0.788). The direct effect and indirect effects of forest cover via PC2 on PCoA2 were also insignificant (direct: coefficient= − 0.145, z= − 0.617, p= 0.537; indirect: coefficient= 0.021, z= − 0.093, p= 0.926). Variables used in this SEM explained 17% of PCoA1 and 3% of PCoA2 (Fig. 5a).

For the rainy season, forest cover had a significant negative effect on PC1, i.e., increased forest cover was associated with decreased PC1 values (sites with more dissolved oxygen) (coefficient= − 0.072, z= − 3.972; p= 0.000; R2 = 0.387). Both direct and indirect effects (via PC1) of forest cover positively affected PCoA1 (direct: coefficient= 0.451, z= 3.853; p= 0.000; indirect: coefficient= 0.332, z= 2.973, p= 0.003). PC1 was negatively associated with PCoA1 (coefficient= − 0.533, z= − 4.561, p= 0.000), but did not affect PCoA2 (coefficient= 0.199, z= 0.837, p= 0.403). This relationship between PCoA1 and PC1 indicated that larger concentrations of dissolved oxygen increased the number of *A. nimbus*, *A. triannulatus*, *A. braziliensis*, *A. peryassui*, whereas greater values of total suspended solids, pH and water temperature increased the number of *A. nuneztovari* and *A. darlingi*. Forest cover did not affect PC2 (coefficient= − 0.005, z= − 0.025, p= 0.980), and PC2 did not affect PCoA1 or PCoA2 respectively (coefficient= 0.063, z= 0.688, p= 0.492; coefficient= − 0.297, z= − 1.597, p= 0.110). The direct effect of forest cover on PCoA2 was also insignificant (direct: coefficient= 0.276, z= 1.163, p= 0.245), as was its indirect effect (indirect: coefficient= − 0.425, z= − 1.498, p= 0.134). Variables used in this SEM explained 79% of PCoA1 and 13% of PCoA2 (Fig. 5b). In summary, pH, dissolved oxygen and total suspended solids (but not temperature) affected anopheline assemblages in both seasons. Forest cover directly and indirectly affected anopheline assemblages in the rainy season, and had a marginal and indirect effect on anopheline assemblages in the dry season.

## Discussion

We untangled how the direct and indirect paths of forest cover and water quality variables interact and shape anopheline assemblages in two seasons. Although previous studies determined how environmental variables at different spatial extents affected anopheline distributions in Amazônia, most studies focused on a single effect of an environmental variable or focused on single habitat types (terrestrial or aquatic)^22,23,41,42^. Our most important finding is that seasonality modulates the direct and indirect effects of forest cover on Amazônian anopheline larval distributions. In particular, we found that forest cover had stronger direct and indirect influence on larval anopheline assemblage composition in the rainy season than the dry season.

The different paths and strengths of forest cover influences on anopheline assemblages during the rainy and dry seasons can be associated with the responses of adults and larvae to forest characteristics. Forest cover influences water quality variables of ponds by shading, organic matter inputs and erosion processes^43^. These effects have consequences for pond water quality^44^ and favor the establishment of different culicid species^45^. We showed that during the rainy season, forest cover directly and indirectly influenced site water quality. Greater forest cover in the rainy season directly and indirectly affected *A. nimbus* and the secondary malaria vectors *A. triannulatus* and *A. braziliensis* positively. In the dry season, greater forest cover positively but marginally affected *A. peryassui*, *A. nuneztovari* and *A. albitarsi*s, but only indirectly through water quality. Some species like *A. triannulatus*, *A. nuneztovari* and *A. braziliensis* coexist with the malaria vector, *A. darlingi,* in breeding sites^46^, and these species have been positively associated with pH, dissolved oxygen and total suspended solids in natural and artificial habitats^20–47^, which are environmental conditions favored by greater forest cover. The marginal indirect effect of forest cover on anopheline assemblage in the dry season suggests that we need caution in the interpretation of this result and long-term temporal data is required to confirm if this effect is corroborated.

Forest conditions influence mosquito vectors and their hosts. For example, some mosquitoes are zoophiles that feed on the blood of birds, reptiles, and mammals^48^, which are often more abundant in conserved areas. Other species are anthropophilic and prefer to feed on human blood^49^ and altered environments can force these species to migrate and, consequently, to change hosts^48^. In our study, *A. triannulatus* and *A. minbus* were more abundant in sites with more natural characteristics, whereas *A. darlingi* and *A. nuneztovar*i were more abundant in altered landscapes. In addition, urbanization and deforestation increase the proximities of humans and domestic animals to mosquito vectors and their hosts, thereby maintaining and increasing transmission cycles^50^.

Forest conditions influence anopheline diversity by different paths, which may alter the strength of their seasonal effects. During the dry season, mosquito survival is also affected by altered microclimate (e.g., lower humidity)^51^ and lentic habitats contain less water, increased nutrient concentrations and decreased abundance and richness of mosquitoes^52,53^. We observed that rainfall plays an important role in the larval abundance of *Anopheles* in artificial larval habitats in Manaus. In addition, climatic factors such as rainfall and river levels are strongly associated with vector abundance and malaria cases in the region^54,55^. During the rainy season, increased water volume in artificial habitats provides more areas for distribution and development of mosquito species^56^ and we detected a significant increase in abundance of *A. triannulatus*, *A. darlingi* and *A. nuneztovari*. These observations may partially explain why we found a direct effect of forest cover on mosquitoes only during the rainy season.

Our results add more evidence that managing and conserving forest cover is important to control anophelines, thereby decreasing the contact of potential vectors (e.g., *A. darlingi*) with humans. In general, our results support the idea that mosquitoes are directly affected by the loss of native forest cover^57^ in the rainy season. Mosquitoes associated with serious human diseases (e.g., malaria, yellow fever, dengue, leishmaniasis) are more abundant in areas with low levels of native forest cover^14–58^. This is a critically important finding because recent studies have shown that forest cover plays an important role in the vector dynamics of mosquitoes and forest conservation keeps pathogens within the forest, avoiding spillover to human settlements^59^. On the other hand, deforestation provides favorable conditions for these vectors, thereby increasing malária cases and decreasing scores of the Human Development Index^60^. In addition, there is a positive correlation between mosquito abundance in fragmented forests and the prevalence of *Plasmodium*, the protozoan that causes malaria^61^.

Artificial larval habitats promote conditions for malaria vectors in Amazônia^62,63^. Therefore, the best way to develop control techniques would be to understand larval ecology in these habitats, where they are more sensitive to infections by pathogens, parasites, predation, larvicides and growth regulators^64^. This information is necessary to minimize failures in programs to control or eradicate the vector and the disease. Under this perspective, our study adds a new piece in the puzzle of mosquito control in Amazônia. For example, during the rainy season when forest cover directly and indirectly influences larval habitats, control programs can strengthen the control of key limnological variables, habitat structure, and entomological aspects, intensifying the environmental filter, particularly in areas with little forest cover and greater human concentrations near those habitats. The limnological study of *Anopheles* larval habitats is still far from complete, as each case has peculiarities inherent to them. Despite attempts, anophelines demonstrate versatility in relation to abiotic parameters^20–26,65,66^. However, we can use approaches that modify the larval environments. For example, more efficient management of water levels in fish farming ponds could decrease larval numbers and anopheline reproduction, Similarly, greater rationing of fish feed would decrease the supply of food resources for mosquito larvae. It is also worth mentioning that some variables are related to the efficiency of others. Regarding biological control via entomopathogenic bacteria, environmental factors (solar radiation) and water quality (amounts of total suspended solids and organic matter), can interfere with the effectiveness of the formulated *Bacillus sphaericus* applied in habitats for vector control^62–67^. Furthermore, eutrophication decreased the assemblages of aquatic invertebrates predating mosquito larvae.

Another alternative is the use of physical control (removal of grasses and macrophytes from the edge of habitats), helping to reduce microhabitats that provide larval refuges. Also, increased light and water temperature at the edges favor natural predation and biological control processes from potential fish and macroinvertebrates. The conservation of natural enemies and the use of biotic agents in the population control of vector mosquitoes have been recommended in small and medium-sized natural and artificial breeding sites^19–53^. A combination of techniques that shape the important environmental variables for the establishment of these species are essential for vector control.

The analytical approach used here opens some windows of opportunity for improvements that are important to be recognized. First, our model did not incorporate important complexity of natural systems, such as ecological interactions among vectors and hosts, including human behavior. Agent-based models, including different host behavior, could provide important insights in this way. Second, our study is very limited in terms of temporal climatic variability. Additional information is needed to better understand the effects of long-term changes in land-use, water quality and climate and their interactions with mosquito assemblages in the region, particularly considering an ecological-evolutionary perspective. Third, it is important to highlight that the magnitude of effects of the estimated drivers were not the same in the rainy and dry seasons. Also, they may not remain constant in coming decades, especially considering potential regional process on mosquito assemblages, such as spillover effects, mass effects and host changes. Fourth, our study was carried out in an area of Amazonia that has experienced, a relatively old land use conversion from forest to urban areas (urban expansion rate of around 12% per year for the past 34 years)^68^. Beginning in the 1970s, human population increased at a rate of around 23% per decade and 25% in Manaus^11^. Therefore, the region we studied is very relevant in terms of historical interactions among human populations, mosquitoes and land use changes. However, understanding the effect of these changes on mosquito assemblages in areas with different land-use change dynamics, provides us with important information^69^, particularly those with very rapid urbanization processes, such as in the Arch of Deforestation^70^. Lastly, we need studies that consider the nexus among climate and land use changes, human and animal population health, economic conditions, and ecosystem services provided by these forest-urban transitional regions. Such information would facilitate including mosquito information in land use planning and climate mitigation programs based on forest management in and around cities.

Therefore, identifying ecological factors and paths that affect the composition of species of epidemiological importance are essential because they inform vector integrated management strategies. We emphasize that larval control in lentic habitats requires knowledge about larval ecology and the effects of biotic and abiotic variables on larvae, especially when it comes to biological controls. The application of integrated pest management can be conducted in both dry and rainy seasons. However, we recommend focusing on the dry season when larval habitats are more limited, in smaller volumes and more accessible for entry and application of vector control techniques. These are critically important considerations because over 2 million people live in Amazonas state^11^ and anophelines transmitted over 59,637 malaria cases in the Amazon region in the first half of 2020, and about 44.4% came from the state of Amazonas^71^.

## Supplementary Information

Supplementary Information.
