# Supplementary material for: Seasonality modulates the direct and indirect influences of forest cover on larval anopheline assemblages in western Amazônia
Source: Sci Rep. 2021 Jun 16;11:12721. doi: 10.1038/s41598-021-92217-9 (PMC8208974; doi:10.1038/s41598-021-92217-9)
Supplement: Supplementary file 1 — Supplementary Information. [file 41598_2021_92217_MOESM1_ESM.docx]

Supplementary Information

Seasonality modulates the direct and indirect influences of forest cover on larval anopheline assemblages in western Amazônia

Adriano Nobre Arcos, Francisco Valente-Neto, Francisco Augusto da Silva Ferreira, Fábio Padilha Bolzan, Hillândia Brandão da Cunha, Wanderli Pedro Tadei, Robert M. Hughes, Fabio de Oliveira Roque.

Supplementary Figure S1. Seasonal variations of water temperature (A), pH (B), dissolved oxygen (C) and total suspended solids (D) in artificial larval habitats.


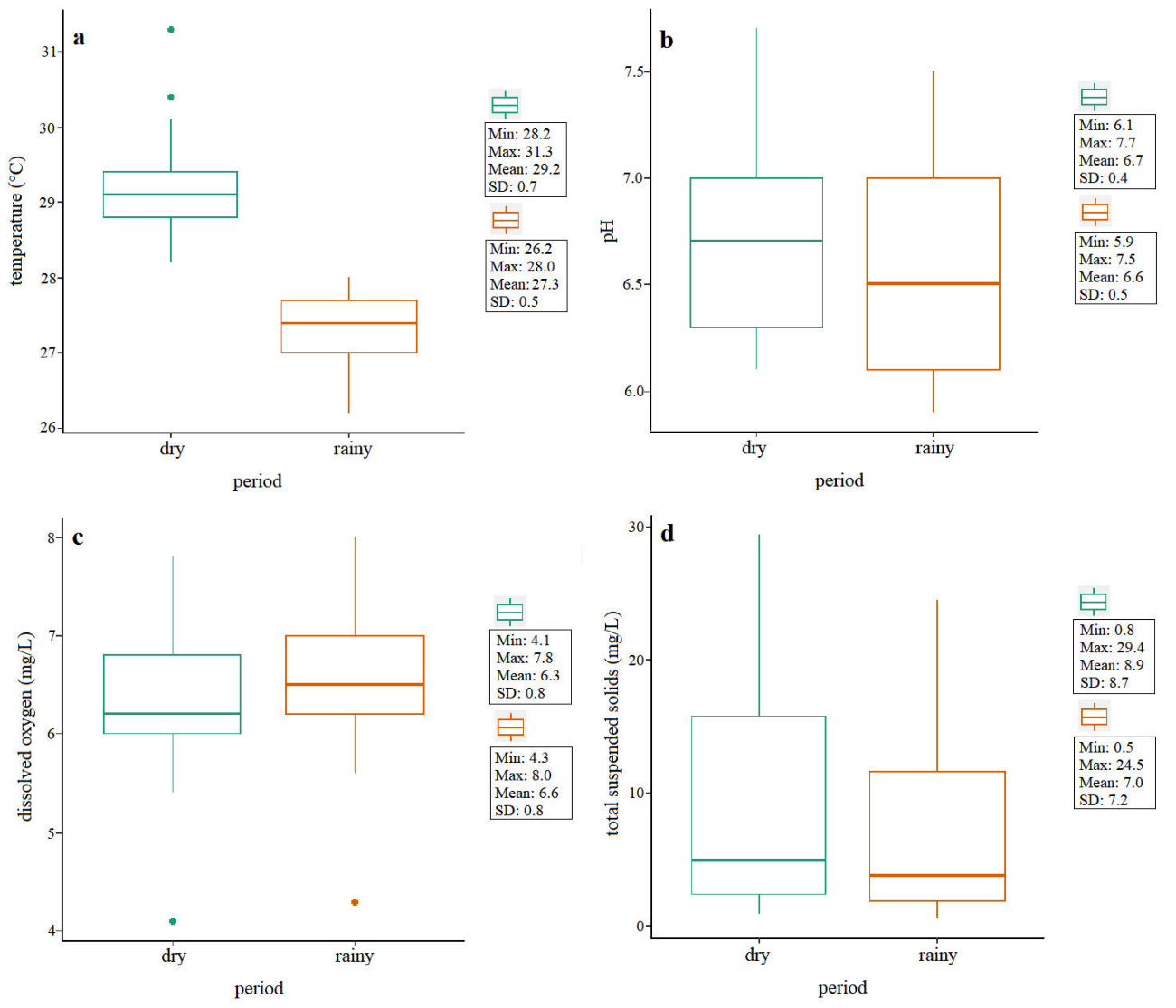


Supplementary Figure S2. Seasonal rainfall amounts and larval abundances in artificial larval habitats.


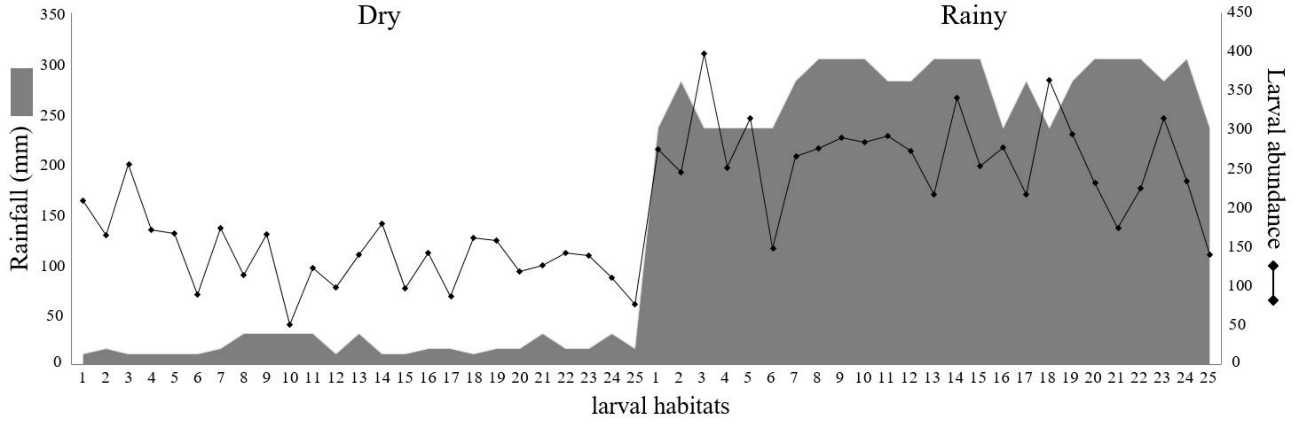


Supplementary Table S1. Seasonal percentages of forest cover and *Anopheles* richness in larval habitats.

| Larval habitat | % Forest Cover | dry | rainy | dry | rainy | dry | rainy | dry | rainy | dry | rainy | dry | rainy | dry | rainy | dry | rainy | dry | rainy | dry | rainy |
| --- | --- | --- | --- | --- | --- | --- | --- | --- | --- | --- | --- | --- | --- | --- | --- | --- | --- | --- | --- | --- | --- |
|  |  | *A. triannulatus* | *A. triannulatus* | *A. darlingi* | *A. darlingi* | *A. nuneztovari* | *A. nuneztovari* | *A. albitarsis* s.l. | *A. albitarsis* s.l. | *A. nimbus* | *A. nimbus* | *A. peryassui* | *A. peryassui* | *A. braziliensis* | *A. braziliensis* | *A. oswaldoi* | *A. oswaldoi* | *A. evansae* | *A. evansae* | *A. deaneorum* | *A. deaneorum* |
| 1 | 86,757 | 156 | 181 | 36 | 71 | 10 | 8 | 1 | 4 | 5 | 6 | 0 | 0 | 2 | 5 | 0 | 0 | 0 | 0 | 0 | 0 |
| 2 | 79,761 | 101 | 126 | 17 | 44 | 20 | 13 | 8 | 11 | 0 | 0 | 9 | 15 | 0 | 0 | 0 | 0 | 0 | 0 | 0 | 0 |
| 3 | 97,630 | 95 | 203 | 57 | 58 | 23 | 19 | 3 | 14 | 74 | 91 | 0 | 2 | 3 | 3 | 1 | 5 | 0 | 2 | 0 | 1 |
| 4 | 87,722 | 66 | 92 | 25 | 41 | 0 | 0 | 9 | 5 | 0 | 11 | 0 | 0 | 0 | 0 | 0 | 0 | 0 | 0 | 0 | 0 |
| 5 | 74,309 | 139 | 239 | 17 | 52 | 0 | 4 | 2 | 14 | 5 | 0 | 0 | 4 | 4 | 0 | 1 | 2 | 0 | 0 | 0 | 0 |
| 6 | 79,830 | 77 | 92 | 26 | 44 | 0 | 10 | 5 | 0 | 0 | 0 | 0 | 3 | 1 | 9 | 0 | 0 | 0 | 0 | 0 | 0 |
| 7 | 74,253 | 123 | 180 | 13 | 28 | 22 | 17 | 6 | 15 | 0 | 2 | 11 | 22 | 0 | 0 | 0 | 0 | 0 | 2 | 0 | 0 |
| 8 | 52,748 | 96 | 120 | 13 | 36 | 0 | 0 | 5 | 9 | 0 | 20 | 0 | 0 | 0 | 0 | 0 | 0 | 0 | 0 | 0 | 0 |
| 9 | 63,132 | 55 | 77 | 55 | 81 | 22 | 18 | 25 | 26 | 4 | 8 | 6 | 10 | 0 | 0 | 0 | 0 | 0 | 0 | 0 | 0 |
| 10 | 48,793 | 10 | 35 | 39 | 44 | 0 | 23 | 2 | 8 | 0 | 0 | 0 | 0 | 0 | 0 | 0 | 0 | 0 | 0 | 0 | 0 |
| 11 | 91,194 | 42 | 74 | 37 | 58 | 11 | 6 | 13 | 17 | 10 | 20 | 8 | 10 | 0 | 2 | 0 | 0 | 2 | 5 | 0 | 0 |
| 12 | 57,142 | 27 | 57 | 29 | 50 | 28 | 22 | 8 | 19 | 3 | 6 | 0 | 0 | 0 | 0 | 3 | 11 | 1 | 8 | 0 | 0 |
| 13 | 92,455 | 59 | 91 | 48 | 64 | 0 | 0 | 1 | 9 | 33 | 44 | 0 | 0 | 0 | 10 | 0 | 0 | 0 | 0 | 0 | 0 |
| 14 | 80,408 | 34 | 103 | 36 | 74 | 19 | 20 | 15 | 33 | 25 | 41 | 18 | 19 | 0 | 0 | 24 | 37 | 9 | 11 | 0 | 3 |
| 15 | 78,263 | 35 | 74 | 47 | 69 | 0 | 0 | 5 | 10 | 0 | 8 | 0 | 16 | 0 | 0 | 0 | 0 | 0 | 0 | 0 | 0 |
| 16 | 82,195 | 19 | 91 | 71 | 104 | 17 | 13 | 2 | 16 | 0 | 0 | 26 | 30 | 8 | 15 | 0 | 0 | 0 | 9 | 0 | 0 |
| 17 | 85,165 | 27 | 88 | 62 | 90 | 30 | 22 | 1 | 10 | 0 | 0 | 18 | 35 | 5 | 11 | 3 | 7 | 0 | 2 | 0 | 0 |
| 18 | 72,079 | 50 | 99 | 71 | 81 | 0 | 29 | 1 | 14 | 42 | 50 | 9 | 16 | 11 | 19 | 0 | 0 | 0 | 3 | 0 | 0 |
| 19 | 82,637 | 89 | 148 | 52 | 94 | 0 | 15 | 3 | 19 | 1 | 0 | 2 | 4 | 4 | 9 | 6 | 2 | 2 | 2 | 0 | 1 |
| 20 | 80,188 | 56 | 126 | 43 | 68 | 9 | 16 | 5 | 5 | 0 | 0 | 6 | 11 | 0 | 2 | 0 | 4 | 0 | 0 | 0 | 0 |
| 21 | 96,931 | 47 | 60 | 65 | 82 | 0 | 0 | 10 | 23 | 5 | 8 | 0 | 0 | 0 | 0 | 0 | 2 | 0 | 0 | 0 | 0 |
| 22 | 81,739 | 77 | 112 | 21 | 57 | 9 | 12 | 8 | 20 | 0 | 0 | 3 | 2 | 3 | 18 | 0 | 0 | 0 | 0 | 0 | 2 |
| 23 | 74,599 | 67 | 134 | 41 | 79 | 0 | 11 | 7 | 31 | 2 | 0 | 1 | 3 | 2 | 5 | 0 | 3 | 0 | 2 | 0 | 0 |
| 24 | 58,963 | 45 | 70 | 57 | 82 | 6 | 16 | 3 | 20 | 0 | 0 | 0 | 0 | 0 | 3 | 0 | 0 | 0 | 0 | 0 | 0 |
| 25 | 82,954 | 24 | 54 | 44 | 62 | 0 | 0 | 3 | 3 | 0 | 0 | 4 | 10 | 2 | 8 | 0 | 2 | 0 | 0 | 0 | 0 |
